# Rare Case of Complete Androgen Insensitivity Syndrome

**DOI:** 10.7759/cureus.54550

**Published:** 2024-02-20

**Authors:** Luís Cesar Fava Spessoto, Júlia Saraiva Avelino Silveira, Andres Menacho Abularach, Gustavo Santana Garcia, Matheus Castro Almeida, Guilherme Cerqueira Gonzales, Ana Clara Nagle Spessoto, Fernando Nestor Facio, Mateus Henrique Silva Faria

**Affiliations:** 1 Urology, Faculty of Medicine of São José do Rio Preto, São José do Rio Preto, BRA; 2 Medicine, Medical School of Catanduva, Catanduva, BRA

**Keywords:** treatment, urology, androgen receptor, male pseudohermaphroditism, androgen insensitivity

## Abstract

Androgen insensitivity syndrome is a rare X-linked recessive condition in which patients present a female phenotype. After complete androgen insensitivity syndrome (CAIS) diagnosis, the timing of gonadectomy should be evaluated, considering the risks and benefits of this procedure. This paper reports an uncommon case of complete androgen insensitivity syndrome diagnosed belatedly in an adult patient. Surgical treatment was deemed necessary due to the elevated risk of gonadal malignancy.

## Introduction

Androgen insensitivity syndrome (AIS) is a rare genetic condition linked to the X chromosome, in which individuals with 46,XY karyotype have a complete (CAIS) or partial (PAIS) impairment of pre- and postnatal virilization and develop a female phenotype [1].

The mutation occurs in the androgen receptor gene, and despite having normal testosterone synthesis, the individual does not develop male characteristics. However, as they have a normal estrogen receptor, the development of female sexual characteristics takes place [2].

Clinical suspicion typically arises during puberty in patients with a female phenotype and primary amenorrhea, reduced pubic and axillary hair, persistent childlike voice, and dyspareunia. Less frequently, it occurs before puberty in cases where patients present with bilateral inguinal hernia, a short vagina, and elevated levels of follicle-stimulating hormone (FSH), luteinizing hormone (LH), and testosterone [3].

Individuals with CAIS present excellent feminization at puberty (normal or augmented breasts, acne-free and body contour) that occurs in response to estrogen produced by testicular and peripheral aromatization of testosterone [4].

In this report, we describe an uncommon case of complete androgen insensitivity syndrome diagnosed belatedly in an adult patient.

## Case presentation

A 33-year-old female patient was referred to the urology outpatient clinic for evaluation due to elevated levels of total testosterone (674 ng/dL) and elongated nodular images adjacent to the ovaries observed in a previous pelvic magnetic resonance imaging (MRI). Additionally, the medical record indicated a karyotype of 46 XY with a provisional diagnosis of Morris syndrome.

**Figure 1:**
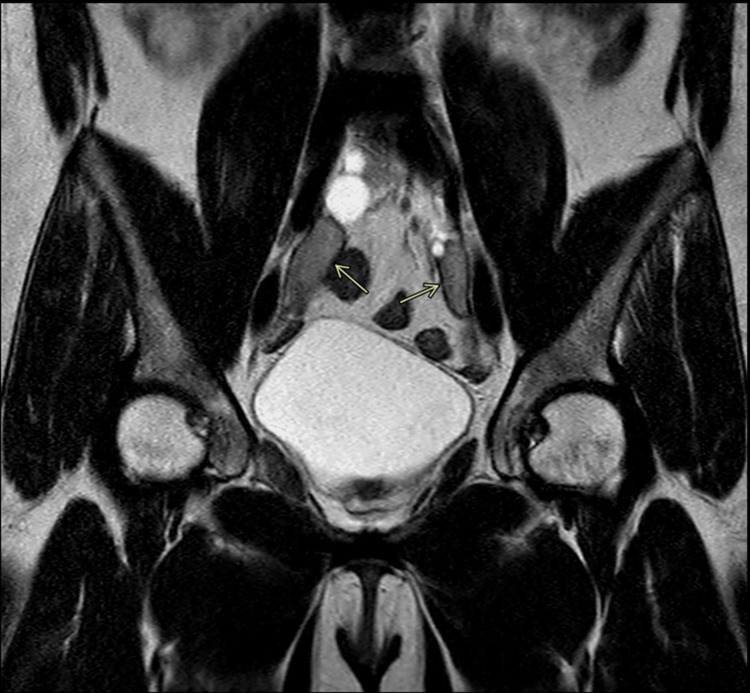
Abdominal MRI revealed oval-shaped, well-defined solid images adjacent to the bilateral iliac vessels, measuring 2.2 x 1.7 cm on the right and 2.4 x 1.0 cm on the left, potentially corresponding to male gonads

Abdominal MRI revealed oval-shaped, well-defined solid images adjacent to the bilateral iliac vessels, measuring 2.2 x 1.7 cm on the right and 2.4 x 1.0 cm on the left, potentially corresponding to male gonads (Figure 1). Adjacent to these images, thin-walled and regular cystic formations are observed, with the largest measuring 1.7 x 1.6 cm on the right, suggesting an association with Müllerian duct cysts located in the pelvic cavity.

**Figure 2:**
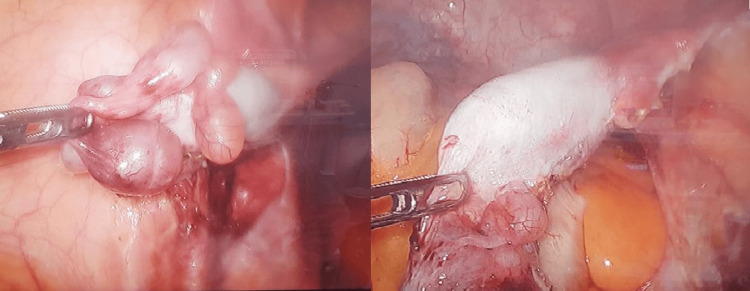
Photograph depicting hypotrophic intra-abdominal testicles

The patient underwent bilateral orchiectomy via videolaparoscopy without complications, revealing hypotrophic intra-abdominal testicles (Figure 2). The patient showed a favorable postoperative course. The anatomopathological result revealed fibrosis and azoospermia.

During outpatient follow-up, the patient initially presented without complaints, but in subsequent visits reported irritability, asthenia, and weight gain. Currently, the patient is undergoing endocrinological evaluation.

## Discussion

CAIS, described by Morris in 1953 as the "feminizing testes syndrome," is a rare recessive genetic disorder linked to the X chromosome. Its prevalence is approximately 1:20,000, and it is considered one of the main causes of disorders of sexual differentiation and the third cause of primary amenorrhea [3].

Until the 6th week of gestation, the embryonic gonad is undifferentiated and bipotential, meaning it contains both sets of internal genital ducts. From that point on, in individuals with normal male sexual differentiation, the testis-determining factor (SRY), present on the Y chromosome, stimulates Leydig cells (LC) to produce testosterone. This hormone acts on the Wolffian ducts (WD), which give rise to the epididymis, vas deferens, and seminal vesicles. Meanwhile, Sertoli cells (SC), under the influence of SRY, stimulate the production of anti-Müllerian hormone, leading to the regression of Müllerian ducts (MD), which would otherwise give rise to female internal organs [4,5].

The differentiation of the genital tubercle into male external genitalia begins between the 9th and 13th weeks of gestation, forming the penis, scrotal sac, and penile urethra. Its development depends on normal levels of testosterone and dihydrotestosterone (DHT), as well as functional hormonal receptors [4,5].

In patients with CAIS, there is a mutation in the androgen receptor gene (AR; X q11-q12), preventing the action of testosterone on the target organs for external male sexual differentiation. This leads to the development of the female phenotype, including the clitoris, small and large labia, and the distal portion of the vagina. It's worth noting that the regression of the Müllerian ducts does not depend on testosterone, and individuals with CAIS will not develop female internal organs (uterus, ovaries, and the upper portion of the vagina).

The clinical presentation and genital phenotype of individuals with CAIS vary, and they may exhibit mild clitoromegaly, partial fusion of the labia minora, genital ambiguity at birth, micropenis, perineal hypospadias, and cryptorchidism. In this case, the patient presented only with hypotrophic intra-abdominal testicles. From 0.8 to 2.4% of patients with a female phenotype and bilateral inguinal hernia present with CAIS [4,6].

Prepubertal diagnosis is challenging and, in most cases, occurs intraoperatively during inguinal hernia repair when the testicle is found. In patients with primary amenorrhea under investigation, imaging studies should be conducted to explore female internal organs, along with karyotyping and investigation of X-chromatin or Y-chromosome [3,4,7]. In this case, CAIS was diagnosed belatedly in an adult patient.

After the diagnosis, the timing of gonadectomy should be evaluated, discussing the risks and benefits of this procedure. In this case, there was a need for hormonal replacement therapy with testosterone due to the symptoms presented by the patient during outpatient follow-up. Considering the high risk of gonadal malignancy (25%) in these cases, surgical treatment in the present report was the best option [8,9].

## Conclusions

Complete androgen insensitivity syndrome is an uncommon condition, and its diagnosis should be made as early as possible due to the risk of gonadal malignancy. Considering that after surgical treatment, hormonal replacement is necessary since the patient will develop hypogonadism.
